# Current standards in the diagnosis and treatment of oral squamous cell carcinoma – a multicenter analysis

**DOI:** 10.3205/iprs000180

**Published:** 2023-10-31

**Authors:** Karsten Schreder, Oliver Thiele, Alexander Eckert

**Affiliations:** 1 University Hospital Halle, University Hospital and Polyclinic of Oral-maxillofacial Surgery, Halle (Saale), Germany; 2 Dental Office, Maxillofacial Surgery, Alexander Raue, Dres. Schäfer, Halle (Saale), Germany; 3 Clinic of Ludwigshafen, Clinic of Oral-maxillofacial Surgery, Ludwigshafen, Germany; 4 Praxis Villa Linhoff, Lippstadt, Germany; 5 Department of Oral and Maxillofacial Plastic Surgery, Paracelsus University Nuremberg, Nuremberg, Germany

**Keywords:** OSCC, German guideline for OSCC, therapeutic options, diagnostic standards, tumor passport, questionnaire

## Abstract

The German guideline for oral squamous cell carcinoma (OSCC) describes the recommended diagnosis and treatment procedures for OSCC and ensures the highest quality patient care. However, the current German guideline for OSCC is indistinct and therapy planning is not standardized in detail between centers. To address this, the current diagnostic and therapeutic strategies in different oral and maxillofacial surgery departments in Germany were summarized using a uniform questionnaire. The results revealed high standards in oncologic maxillofacial care, but non-uniform standards exist between centers. Moreover, an increasing use of diagnostic and treatment methods that are not included in the German guideline for OSCC, such as positron emission tomography computed tomography (PET-CT) and tumor biomarkers, were used by different centers. These results support the updated German guideline for OSCC but highlight the need to consider other additive methods to improve patient care and outcomes. Furthermore, a recommendation to introduce tumor passports to simplify OSCC diagnosis and treatment should be discussed. These changes will improve the prognosis and quality of life of patients with OSCC.

## Abstract (de)

Die deutsche Leitlinie zum oralen Plattenepithelkarzinom beschreibt die empfohlenen Diagnose- und Behandlungsverfahren und gewährleistet eine qualitativ hochwertige Patientenversorgung. Allerdings ist die aktuelle Leitlinie zum oralen Plattenepithelkarzinom unklar und die Therapieplanung nicht im Detail zwischen den Zentren standardisiert. Um diesem Problem Rechnung zu tragen, wurden die aktuellen Diagnose- und Therapiestrategien in verschiedenen Abteilungen der Mund-, Kiefer- und Gesichtschirurgie in Deutschland anhand eines einheitlichen Fragebogens zusammengefasst. Die Ergebnisse zeigten hohe Standards in der onkologischen Betreuung, es bestehen jedoch uneinheitliche Standards zwischen den Zentren. Darüber hinaus wurden in verschiedenen Zentren zunehmend Diagnose- und Behandlungsmethoden eingesetzt, die nicht in der Leitlinie für orale Plattenepithelkarzinome enthalten sind, wie z.B. die Positronenemissionstomographie in Kombination mit der Computertomographie (PET-CT) und Tumorbiomarker. Diese Ergebnisse unterstützen die aktualisierte Leitlinie für orale Plattenepithelkarzinome, unterstreichen jedoch die Notwendigkeit, andere additive Methoden in Betracht zu ziehen, um die Patientenversorgung und die Ergebnisse zu verbessern. Darüber hinaus sollte eine Empfehlung zur Einführung von Tumorpässen zur Vereinfachung der oralen Plattenepithelkarzinom-Diagnose und -Behandlung diskutiert werden. Diese Änderungen werden die Prognose und Lebensqualität von Patienten mit oralen Plattenepithelkarzinom verbessern.

## Introduction

Oral squamous-cell carcinoma (OSCC) is one of the most frequent human malignant cancers worldwide and is a serious and life-threatening disease [1]. Both OSCC and its treatment can cause functional and aesthetic deficits, which can impair quality of life. The recommended diagnosis and treatment of OSCC is described in the German guideline for OSCC [2]. This guideline provides the basis for clear, treatment-relevant decision-making processes and helps to ensure that the OSCC treatments offered to patients are supported by scientific evidence. However, despite this guideline, it is not clear how much treatment strategies differ between craniomaxillofacial surgery clinics and how treatment quality is assessed. To address this, it was the aim of the present investigation to compare treatment and quality control methods used among specialist clinics registered with the German Society of Craniomaxillofacial Surgery using a questionnaire sent to all departments and clinics. Additionally, the use of biomarkers that are currently not included in the updated German guideline for oral squamous cell carcinoma was also examined.

## Material and methods

All departments registered with the Deutsche Gesellschaft für Mund-, Kiefer- und Gesichtschirurgie (German Society of Craniomaxillofacial Surgery) were contacted. E-mails were sent to the head of each department with a link to the survey. The survey was developed using EvaSys^®^ professional survey software. The questionnaire (Attachment 1) evaluated the diagnostic and therapeutic strategies used by individual clinics for OSCC. The questionnaire was divided into three subunits. The first subunit covered general information about the clinic, including whether it contained a certified tumor center and whether tumor passports were used. The second subunit covered scientific-translational information such as the use of tumor markers and novel therapeutic approaches not currently listed in the German guideline for OSCC (immune checkpoint inhibitors, antibody therapy). Particularly important here were the pre- and post-therapeutic imaging diagnostic techniques used. The third subunit covered patient follow-up methods, such as tumor dispensary consultations and palliative care.

Six months after the initial e-mail was sent out, the results were collected and evaluated. Completed questionnaires were analyzed using SPSS 26.5^©^.

## Results

The questionnaire was sent to 81 departments of oral and maxillofacial surgery in Germany and 32 departments responded. Of these, 30 were fully evaluable, giving a response rate of 37%. 50% of the completed questionnaires were returned from university hospitals (15) and 50% were returned from non-university hospitals (15). Results from the first subunit showed that less than 33% of the responding clinics used a tumor passport. In the case of such a passport, 40% correspond to a use in a general version, not subject-specific for head and neck/oropharynx tumors, 60% of the clinics used requirements specifically for head and neck/oropharynx tumors, such as those already used in ear-nose and throat medicine, but not especially for OSCC.

**Figure 1:**
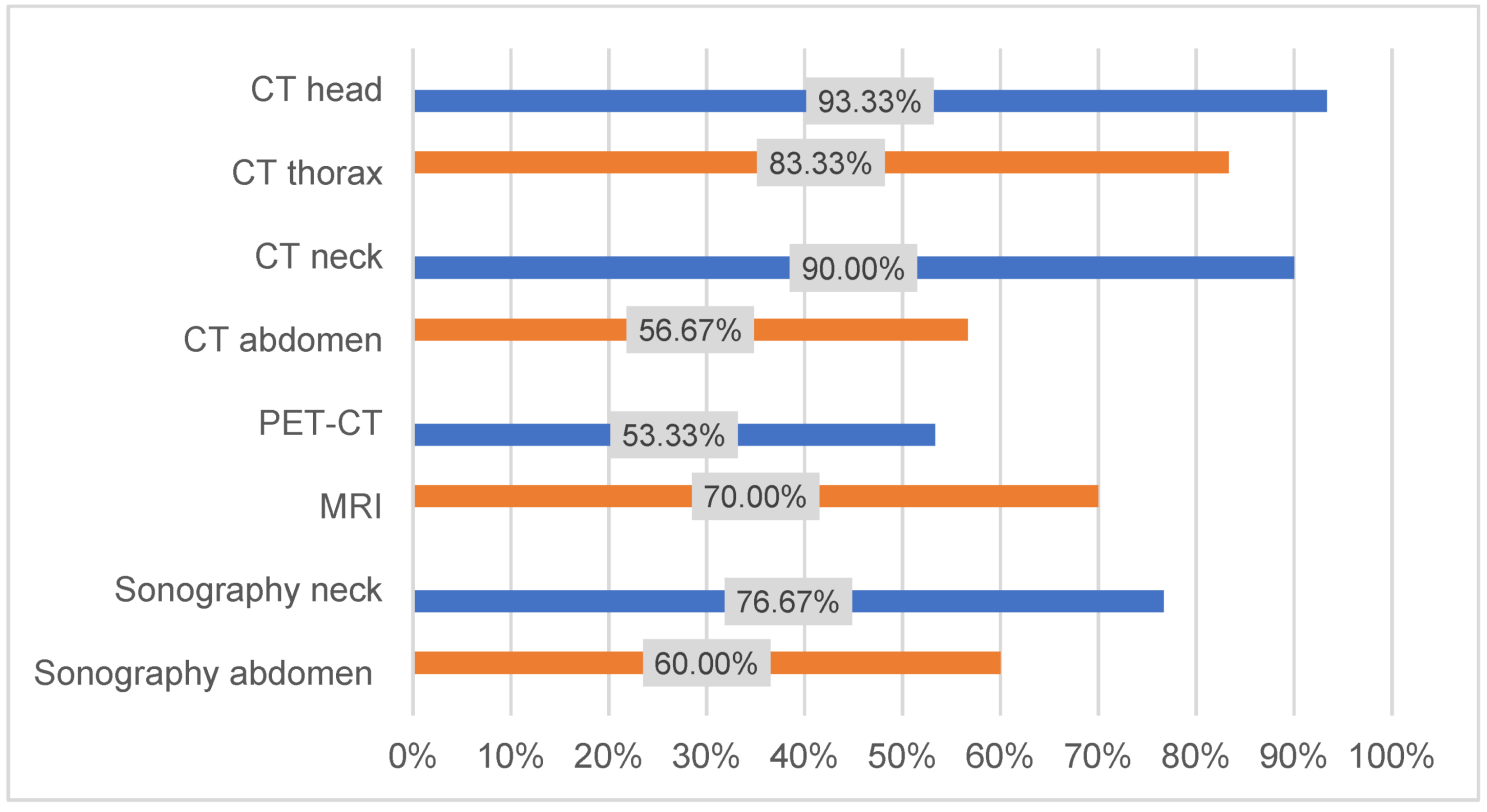
Preoperative diagnostic staging (n=30)

60% of the responding clinics contained a head and neck tumor center that was certified by the German Society of Craniomaxillofacial Surgery. Differences in preoperative staging, perioperative therapy, and postoperative follow-up were also found (Figure 1 (Fig. 1)). A lack of standardized guidance in the German guideline for OSCC might explain these differences. 

Results from the second subunit showed that preoperative staging was similar between centers. More than 90% used a head and neck CT, 83% used a chest CT, and 70% used magnetic resonance imaging in preoperative staging (Figure 1 (Fig. 1)).

**Figure 2:**
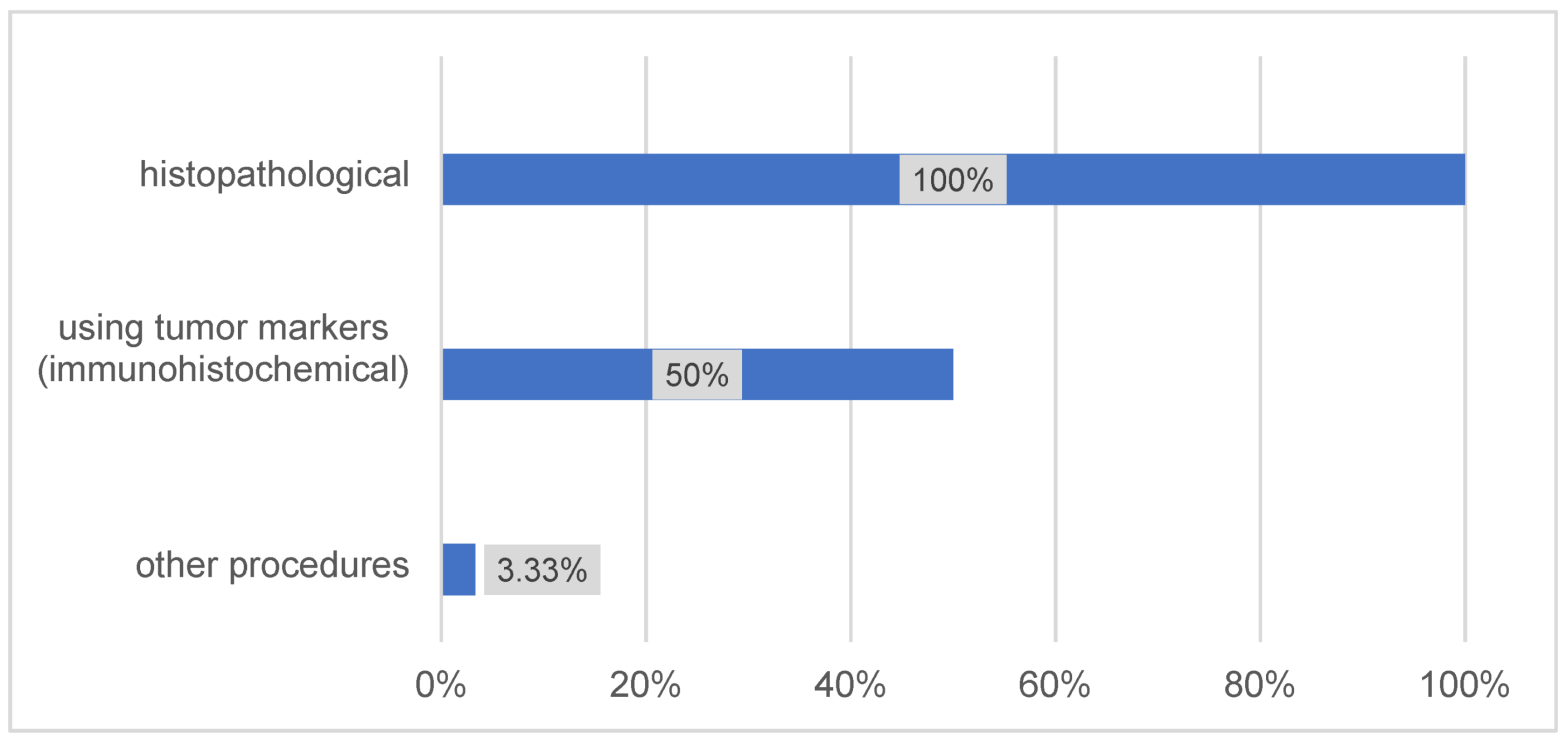
Preoperative tumor staging techniques (n=30)

**Figure 3:**
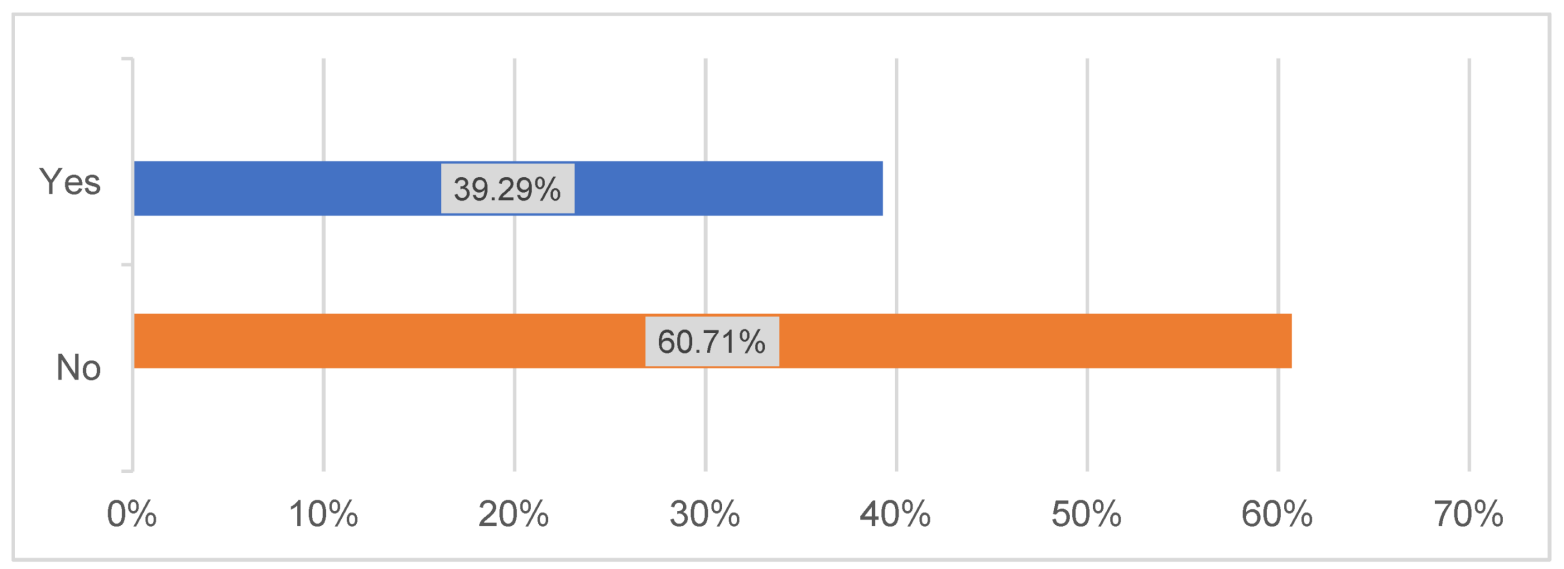
Advanced/escalating therapeutic approaches (n=28)

Moreover, PET-CT was routinely performed in 53% of centers. Regarding tumor markers, 50% of the responding centers indicated that they already use additive tumor markers for preoperative tumor staging (Figure 2 (Fig. 2)). In addition, 61% of the responding clinics reported using novel therapeutic approaches that are not described in the current German guideline for OSCC (Figure 3 (Fig. 3)).

Participating centers also indicated that they were using therapeutic approaches that are not currently described in the German guideline for OSCC, including antibodies or other immunological strategies such as immune checkpoint inhibitors, photodynamic therapies, arterial hemoperfusion, or using a cyber knife. Salvage surgery and established chemotherapy techniques were also consistently reported. 

**Figure 4:**
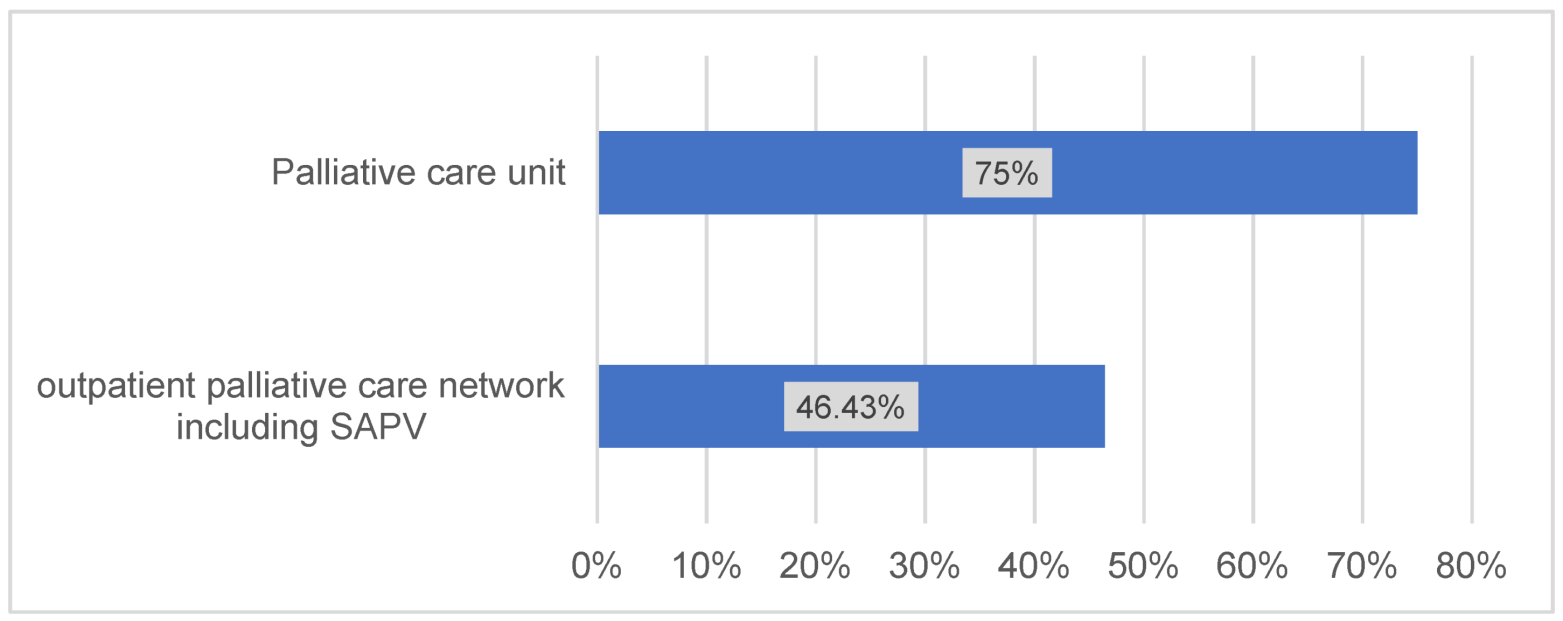
Palliative supply system in German craniomaxillofacial clinics (n=27)

In the third subunit of the questionnaire, more than 92% of the participating clinics reported using a special tumor dispensary and palliative care. Palliative care units were indicated for 75% of the centers and 47% offered a kind of specialized palliative home care (in German so-called spezialisierte ambulante Palliativversorgung SAPV) (Figure 4 (Fig. 4)).

## Discussion

In this study, the diagnostics, therapeutic strategies, and patient follow-up between OSCC clinics in Germany were examined and compared. It shows that OSCC diagnostic and treatment procedures differ between centers at a high level of diagnostic and therapeutic standard, highlighting a need to standardize and improve the German guideline for OSCC [2]. 

Some aspects should be discussed more in detail. These are the role of panendoscopy, scoring for an individualized therapy and additional aspects in immunotherapy. First, the essential role of panendoscopy for staging in OSCC patients has been extensively discussed in numerous articles. For example, Metzger and coworkers described the impact of this technology in reliably detecting synchronous malignancies in the head and neck region [3]. Moreover, Spoerl et al. [4] evaluated the efficiency of panendoscopy to examine the prevalence of synchronous upper aerodigestive tract (UAT) tumors within OSCC patients . As a result, a second UAT tumor in OSCC patients correlated with survival data. Both, the overall survival (63.9% vs 43.5%, p=0.010) as well as recurrence free survival (57.1% vs 32.4%, p=0.016) decreased [4]. In contrast to this results, routine panendoscopy was not recommended in all OSCC patients. The authors concluded that there was no general recommendation, especially not in low-risk oral cancer patients like non-smokers and non-drinkers by Valentin et al. [5]. In general, it can be formulated that pandendoscopy should be considered as an essential standard procedure in the diagnosis of an OSCC. Second, two indices are of crucial interest for an individualized therapy decision in the context of the tumor board: Tumor Proportion Score (TPS) and Combined Positive Score (CPS). Both are used in the case of programmed cell death ligand 1 (PD-L1) – one of the key steps in promoting immune resistance may help to characterize OSCC. As a consequence, and third, PD-L1 may be considered as additional prognostic biomarker [6]. In addition, it is assumed, that this above-mentioned PD-L1-scoring is helpful for further therapeutical decisions. Kitichotkul and coworkers postulated that approximately one-fourth of OSCC cases are PD-L1-positive, suggesting candidacy for anti-PD-L1 immunotherapy [7]. In this analysis, the authors found that PD-L1 expression was positive in 25.9% of all cases at CPS≥1. Therefore, this subpopulation of OSCC is eligible to immunotherapy targeting PD-L1 [7], [8]. Both indices are of great interest to maximize the benefit of blockade PD-1/PD-L1 axis and may act as an effective predictor before starting with additive immune therapeutical strategies like PD-1/PD-L1-inhibitors [9]. To sum up, both PD-1 and PD-L1 should be routinely analyzed in each OSCC case. 

Targeted collection of patient data from different clinics can provide an overview of the treatment level and established standards. Earlier multicenter studies have used questionnaires and interviews to determine which cleft or orthognathic surgery techniques are used in maxillofacial clinics across Germany [10], [11] and identified those treatments approaches that gave the best patient outcomes. The present study is the first to use a similar multicenter approach to investigate diagnosis and treatment of OSCC in Germany. So far, data on this topic has been limited to investigations of treatment strategies in individual clinics and no reports have been made on whether these treatments correspond with the German guideline for OSCC. The present study addresses these gaps in the current knowledge.

Electronic tumor passports have been evaluated in the literature for pediatric cancers [12] such as medulloblastoma [13], but not for OSCC so far. This study shows that some centers are using tumor passports for tumors in the head and neck/oropharynx region, but not specifically for OSCC. Given the rising incidence of OSCC, it may be necessary to consider using tumor passports for OSCC in the future to simplify preoperative planning and improve patient care. 

The results showed that more and more centers are using novel diagnostic and therapeutic strategies to deal with OSCC, highlighting a growing need to incorporate these emerging techniques into the German guideline for OSCC. Biomarkers [14] and genomic and epigenetic signatures [15] have been used to successfully diagnose and characterize oral cancers, so biomarkers and signature proteins should be incorporated into the German guideline for OSCC. Other promising strategies include immune checkpoint blockade, photodynamic therapies, arterial hemoperfusion, hypoxia-dependent and hypoxia-independent changes in metabolic adaption, and immune monitoring. In addition, tumors signatures can be characterized using transcription variants and may represent important prognostic factors for OSCC. The results showed that current approaches to postoperative follow-up of OSCC patients are satisfactory in Germany. However, we identified palliative treatment as a potential area for further consideration, particularly whether palliative care should be provided at home or in the palliative care unit.

## Conclusion

To sum up, the results revealed non-uniform but excellent oncological care in centers in Germany. The German guideline for OSCC [2] represents high standards for the diagnosis and treatment of OSCC. However, the findings show that this guideline could be improved by standardizing certain procedures and including novel diagnostic and therapeutic techniques. Diagnostic techniques to add to the German guideline for OSCC include PET-CT scanning (although this is expensive so may not always be feasible) and tumor markers. Furthermore, the use of biomarkers to diagnose OSCC should be standardized and a uniform OSCC passport should be introduced. This will simplify preoperative diagnostics by specifying the type of tumor and the course of previous illness. These improvements to the German guideline for OSCC will optimize patient care and maximize quality of life.

## Notes

### Acknowledgements

The authors thank the participants of the survey (see Attachment 2). 

### Competing interests

The authors declare that they have no competing interests. 

### Authors’ contributions

Karsten Schreder and Oliver Thiele contributed equally to the manuscript. 

## Supplementary Material

Questionnaire

Participants of the survey
