# Supplementary material for: Current standards in the diagnosis and treatment of oral squamous cell carcinoma – a multicenter analysis
Source: GMS Interdiscip Plast Reconstr Surg DGPW. 2023 Oct 31;12:Doc10. doi: 10.3205/iprs000180 (PMC10666175; doi:10.3205/iprs000180)
Supplement: Questionnaire [file IPRS-12-10-s-001.pdf]

SurveyGrid	Survey in cranio-maxillofacial-surgery as a part of a doctoral thesis	Electric Paper

Please mark this way: ☐ ☒ ☐ ☐ ☐
correction: ☐ ■ ☐ ☒ ☐ In the interest of optimal data collection, please note the information given on the left when filling out the form.

1. General

1.1 Please enter your postcode: (to check the response rate)

2. General tumor passport

2.1 Do you use a tumor pass? ☐ YES ☐ NO

2.2 If yes: ☐ general execution ☐ especially for head and neck/ oropharyx tumors

3. General tumor center

3.1 Is it a head and neck tumor center certified by the German Cancer Society? ☐ YES ☐ NO

4. Preoperative staging (multiple answers possible)

Which diagnostics do you use?

☐ CT head ☐ CT chest ☐ CT neck
☐ CT abdomen ☐ PET-CT ☐ MRI
☐ neck sonography ☐ abdomen sonography

4.2 Trial backup

☐ histopathological ☐ using tumor markers (immunhistochemical) ☐ other procedures

4.3 if other procedures, which?

5. (peri)operatively

5.1 Therapy according to the german guidlines for oral-squamous-cell-carcinoma? ☐ YES ☐ NO

5.2 since when have you been working according to the guidelines? (please indicate the year)

5.3 further / escalating therapeutic approaches? ☐ YES ☐ NO

5.4 if YES, which?:

6. postoperative follow-up care (multiple answers possible)

Attachment 1 to: Schreder K, Thiele O, Eckert A. Current standards in the diagnosis and treatment of oral squamous cell carcinoma – a multicenter analysis. GMS Interdiscip Plast Reconstr Surg DGPW. 2023;12:Doc10. DOI: 10.3205/iprs000180

6. postoperative follow-up care (multiple answers possible) [continuation]

6.1 Re-Staging

- ☐ CT head
- ☐ CT thorax
- ☐ CT neck
- ☐ CT abdomen
- ☐ PET-CT
- ☐ MRI
- ☐ neck sonography
- ☐ abdomen sonography

7. Postoperative follow-up intervals

(please enter the number in the free box and examinations; multiple answers: - "- (quotation marks) possible)

7.1 monthly in / from the .... th year after the initial diagnosis

7.2 what investigation?:

7.3 every two months in / from the .... th year after the initial diagnosis

7.4 what investigation?:

7.5 quarterly in / from the .... th year after the initial diagnosis

7.6 what investigation?:

7.7 every six months in / from the .... th year after the initial diagnosis

7.8 what investigation?:

7.9 annually in / from the .... th year after the initial diagnosis

7.10 what investigation?:

7.11 Do you have a special tumor dispensary consultation hour? ☐ YES ☐ NO

8. Palliative care

8.1 ☐ YES ☐ NO

Attachment 1 to: Schreder K, Thiele O, Eckert A. Current standards in the diagnosis and treatment of oral squamous cell carcinoma – a multicenter analysis. GMS Interdiscip Plast Reconstr Surg DGPW. 2023;12:Doc10. DOI: 10.3205/iprs000180

8. Palliative care (continuation)

8.2 if YES, to what extent ?: (palliative ward, outpatient palliative network, ...)

8.3 Do you have palliative care cooperation partners ?: ☐ YES ☐ NO

8.4 if YES, which?:

Attachment 1 to: Schreder K, Thiele O, Eckert A. Current standards in the diagnosis and treatment of oral squamous cell carcinoma – a multicenter analysis. GMS Interdiscip Plast Reconstr Surg DGPW. 2023;12:Doc10. DOI: 10.3205/iprs000180
