# Supplementary material for: Current standards in the diagnosis and treatment of oral squamous cell carcinoma – a multicenter analysis
Source: GMS Interdiscip Plast Reconstr Surg DGPW. 2023 Oct 31;12:Doc10. doi: 10.3205/iprs000180 (PMC10666175; doi:10.3205/iprs000180)
Supplement: Participants of the survey [file IPRS-12-10-s-002.pdf]

Participants of the survey

Clinic	Contact
Johannes-Gutenberg-University of Mainz, Clinic of Oral-maxillofacial Surgery, Mainz, Germany	Bilal Al-Nawas
HELIOS Clinic Berlin-Buch, Clinic of Oral-maxillofacial Surgery, Berlin, Germany	Reinhard Bschorer
Clinic of Bremerhaven Reinkenheide gGmbH, Clinic of Oral-maxillofacial Surgery, Bremerhaven, Germany	André Eckardt
Medical School of Hannover, Clinic and Polyclinic of Oral-maxillofacial Surgery, Hannover, Germany	Nils-Claudius Gellrich
Witten/Herdecke University, Dortmund General Hospital, Clinic of Oral-maxillofacial Surgery, Dortmund, Germany	Stefan Haßfeld
Charité – Universitätsmedizin Berlin, corporate member of Freie Universität Berlin and Humboldt-Universität zu Berlin, Department of Oral and Maxillofacial Surgery, Berlin, Germany	Max Heiland
University Hospital Leipzig, Clinic of Oral-maxillofacial Surgery, Leipzig, Germany	Alexander Hemprich
University Hospital Heidelberg, Dept. of Oral and Maxillofacial Surgery, Heidelberg, Germany	Jürgen Hoffmann
University Hospital of RWTH Aachen, Clinic and Polyclinic of Oral-maxillofacial Surgery, Aachen, Germany	Frank Hölzle
DONAUISAR Clinic of Oral-maxillofacial Surgery, Deggendorf, Germany	Cornelius Klein
Specialist Clinic Hornheide, Department of Oral-maxillofacial Surgery, Münster, Germany	Martin Klein
University Hospital Würzburg, Clinic and Polyclinic of Oral-maxillofacial Surgery, Würzburg, Germany	Alexander Kübler
University Hospital of Technical University of Dresden „Carl-Gustav-Carus“, Clinic and Polyclinic of Oral-maxillofacial Surgery, Dresden, Germany	Günter Lauer
HELIOS Clinic of Erfurt, Clinic of Oral-maxillofacial Surgery, Plastic Surgery, Erfurt, Germany	Jörn-Uwe Piesold
University of Bonn, Clinic and Polyclinic of Oral-maxillofacial Surgery, Bonn, Germany	Rudolf Reich
Hospital of the University of Regensburg, Clinic and Polyclinic of Oral-maxillofacial Surgery, Regensburg, Germany	Torsten Reichert
University Hospital of Tübingen, Clinic and Polyclinic of Oral-maxillofacial Surgery, Tübingen, Germany	Siegmar Reinert
Clinic of Saarbrücken, Oral-maxillofacial Surgery and Plastic Surgery, Saarbrücken, Germany	Herbert Rodemer
Evangelic Hospital Bethesda Mönchengladbach GmbH, Oral-maxillofacial Operations, Möchengladbach, Germany	Daniel Rothamel
Clinic Centre of Bremen-Mitte, Department of Oral and Maxillofacial Surgery, Bremen, Germany	Jan Rustemeyer
Clinic of Johann-Wolfgang-Goethe-University, Clinic and Polyclinic of Oral-maxillofacial Surgery and Plastic Surgery, Frankfurt, Germany	Robert Sader
Diaconal Hospital Rotenburg (Wümme) gGmbH, Clinic of Oral-maxillofacial Surgery, Rotenburg (Wümme), Germany	Christian Schippers
University Medicine Göttingen, Georg-August-University, Center of Tooth-mouth-orthodontics, Department of Oral-maxillofacial Surgery, Göttingen, Germany	Henning Schliephake
RoMed Clinic, Clinic of Oral-maxillofacial Surgery, Rosenheim, Germany	Gregor Schmidt-Tobolar
University Hospital Jena, Clinic and Polyclinic of Oral-maxillofacial Surgery / Plastic Surgery, Jena, Germany	Stefan Schultze-Mosgau
Clinic of Ernst von Bergmann Potsdam gGmbH, Head and Skin Center, Clinic of Oral-maxillofacial Surgery, Potsdam, Germany	Thomas Teltzrow
Red Cross Hospital Kassel nonprofit GmbH, Clinic of Oral-maxillofacial Surgery, Kassel, Germany	Hendrik Terheyden

Attachment 2 to: Schreder K, Thiele O, Eckert A. Current standards in the diagnosis and treatment of oral squamous cell carcinoma – a multicenter analysis. GMS Interdiscip Plast Reconstr Surg DGPW. 2023;12:Doc10. DOI: 10.3205/iprs000180
